# {*S*-Benzyl 3-[(6-methyl­pyridin-2-yl-κ*N*)methyl­idene]dithio­carbazato-κ^2^ *N* ^3^,*S*}zinc

**DOI:** 10.1107/S1600536812013529

**Published:** 2012-04-04

**Authors:** Thahira B. S. A. Ravoof, Siti Aminah Omar, Mohamed Ibrahim Mohamed Tahir, Karen A. Crouse

**Affiliations:** a Department of Chemistry, Faculty of Science, Universiti Putra Malaysia, 43400 UPM, Serdang, Selangor, Malaysia

## Abstract

The title compound, [Zn(C_15_H_14_N_3_S_2_)_2_], contains two chemically equivalent Schiff base anions that are coordinated to the Zn^II^ ion as tridentate *N*,*N*′,*S*-chelating ligands, creating a distorted octa­hedral environment [the smallest angle being 75.40 (6)° and the widest angle being 162.87 (6)°], with the two S atoms in *cis* positions. The dihedral angle between the mean planes of the two coordinating ligands is 85.65 (5)°. Weak C—H⋯S hydrogen bonds are also observed.

## Related literature

For background to the coordination chemistry of hydrazine carbodithio­ates, see: Ravoof *et al.* (2010 ▶). For the synthesis, see: Ali *et al.* (1997 ▶); Ravoof *et al.* (2004 ▶). For related structures, see: Ali *et al.* (2001 ▶); Tarafder *et al.* (2001 ▶).
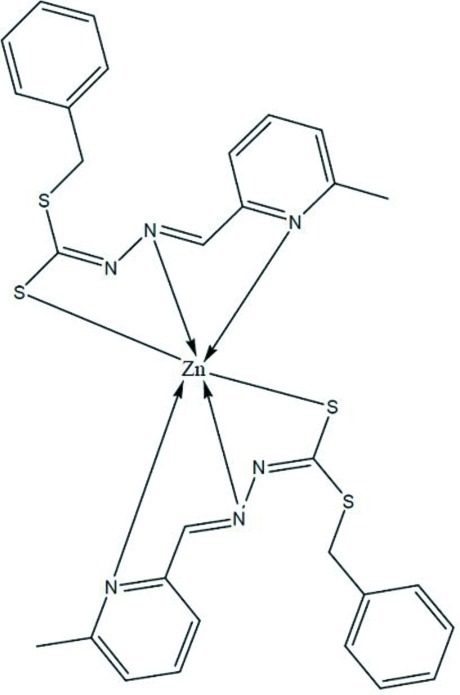


## Experimental

### 

#### Crystal data

[Zn(C_15_H_14_N_3_S_2_)_2_]
*M* *_r_* = 666.24
Monoclinic, 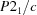

*a* = 14.8931 (8) Å
*b* = 13.0630 (5) Å
*c* = 17.1706 (9) Å
β = 112.855 (6)°
*V* = 3078.2 (3) Å^3^
*Z* = 4
Mo *K*α radiation
μ = 1.10 mm^−1^
*T* = 150 K
0.24 × 0.18 × 0.16 mm

#### Data collection

Oxford Diffraction Gemini diffractometer
Absorption correction: multi-scan (*CrysAlis PRO*; Agilent, 2011 ▶) *T*_min_ = 0.82, *T*_max_ = 0.84
20496 measured reflections
7140 independent reflections
5960 reflections with *I* > 2.0σ(*I*)
*R*_int_ = 0.040

#### Refinement

*R*[*F* ^2^ > 2σ(*F* ^2^)] = 0.035
*wR*(*F* ^2^) = 0.076
*S* = 1.00
7140 reflections
370 parameters
H-atom parameters constrained
Δρ_max_ = 0.60 e Å^−3^
Δρ_min_ = −0.44 e Å^−3^

### 

Data collection: *Gemini User Manual* (Oxford Diffraction, 2006 ▶); cell refinement: *CrysAlis PRO* (Agilent, 2011 ▶); data reduction: *CrysAlis PRO*; program(s) used to solve structure: *SIR92* (Altomare *et al.*, 1994 ▶); program(s) used to refine structure: *CRYSTALS* (Betteridge *et al.*, 2003 ▶); molecular graphics: *CAMERON* (Watkin *et al.*, 1996 ▶); software used to prepare material for publication: *CRYSTALS*.

## Supplementary Material

Crystal structure: contains datablock(s) global, I. DOI: 10.1107/S1600536812013529/wm2612sup1.cif

Structure factors: contains datablock(s) I. DOI: 10.1107/S1600536812013529/wm2612Isup2.hkl

Additional supplementary materials:  crystallographic information; 3D view; checkCIF report

## Figures and Tables

**Table 1:** Selected bond lengths (Å)

Zn1—N102	2.1032 (17)
Zn1—S105	2.4885 (6)
Zn1—N115	2.2692 (16)
Zn1—N202	2.1005 (16)
Zn1—S205	2.5263 (6)
Zn1—N215	2.2117 (17)

**Table 2:** Hydrogen-bond geometry (Å, °)

*D*—H⋯*A*	*D*—H	H⋯*A*	*D*⋯*A*	*D*—H⋯*A*
C217—H2171⋯S105^i^	0.95	2.82	3.697 (3)	155

Symmetry code: (i) 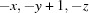.

**Fig. 1.**
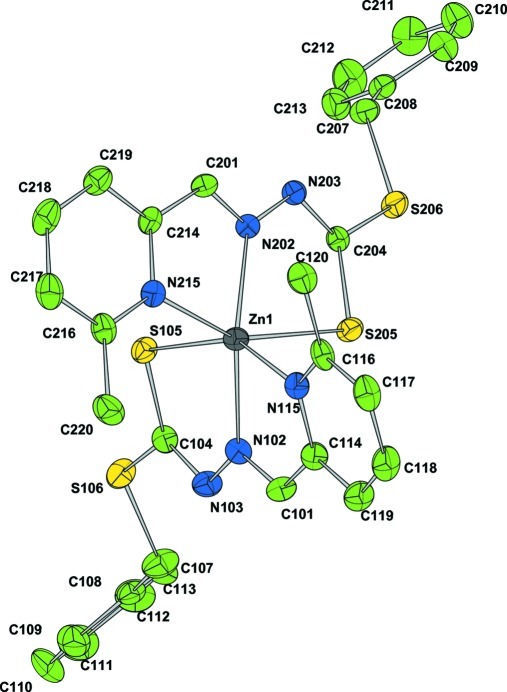
The title compound with displacement ellipsoids drawn at the 50% probability level. H atoms are omitted for clarity.

**Fig. 2.**
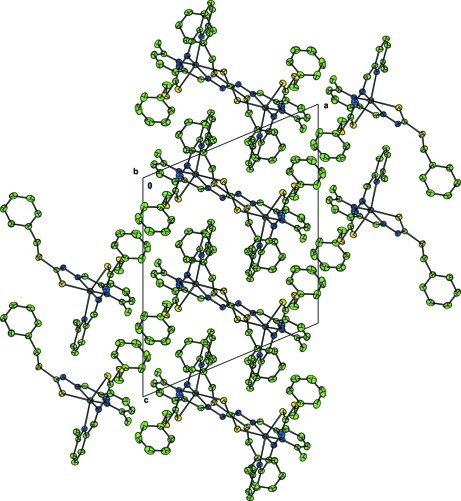
Molecular packing diagram of the title compound viewed along the b axis. Hydrogen atoms are omitted for clarity.

## supplementary crystallographic information

### Comment

The title compound was preferentially formed during the attempt to complex the tridentate Schiff base with zinc(II) saccharinate. The saccharinate anion was eliminated in this process and two tridentate deprotonated Schiff base moieties coordinated instead with the zinc(II) cation as determined by its infrared spectrum, elemental analysis and crystal structure analysis.

The bidentate ligands coordinate through their pyridine nitrogen, azomethine nitrogen and thiolate sulfur atoms. The environment around the Zn^II^ ion is distorted octahedral (Fig. 1) with N—Zn—N, N—Zn—S and S—Zn—S angles between 75.40 (6)° and 162.87 (6)°. The deprotonation of the ligands is accompanied by their tautomerism to the iminothiolate forms. While coordinating in the iminothiolate form, the negative charge generated upon deprotonation is delocalized in the C—N—N—C system as observed by their intermediate bond lengths: C104—N103 = 1.311 (3) Å, N103—N102 = 1.384 (2) Å, N102—C101 = 1.276 (3) Å and C204—N203 = 1.311 (3) Å, N203—N202 =1.380 (3) Å, N202—C201 =1.281 (2) Å. Similar bond lengths and angles have been observed in the octahedral zinc(II) complex containing the pyridine-2-aldehyde Schiff base of *S*-benzyldithiocarbazate (Tarafder *et al.*, 2001) for which C—N bond lengths ranging from 1.273 to 1.320 Å and N—N bond lengths of 1.374 to 1.379 Å were reported. In the title complex, the two ligands are coordinated to the zinc(II) ion in a meridional configuration, where the two thiolate S atoms (S105 & S205) and the two pyridine N atoms (N115 & N215) are *cis* to each other and the two azomethine N atoms *trans* (N102 & N202) as in other bis-ligand metal complexes of related NNS tridentate ligands (Tarafder *et al.*, 2001). The angle between the planes defined by Zn1—S205—C204—N203—N202—C201—C214—N215 (minimum deviation: 0.001 and maximum deviation: 0.157 Å), and Zn1—S105—C104—N103 –N102—C101—C114—N115 (minimum deviation: 0.011 and maximum deviation: 0.212 Å) is 85.65 (5)° showing that the planes are almost orthogonal to each other thus defining an distorted octahedral arrangement.

The angle between the planes defined by the benzyl ring (C208—C213) and the pyridyl ring (C214—C219) is 84.39 (12)°; however, the angle between the planes defined by the corresponding benzyl (C108—C113) and pyridyl rings (C114—C119) in the other Schiff base moiety is 75.06 (12)°. Both planes of the benzyl ring moieties of the Schiff bases are slightly displaced at an angle of 15.05 (10)°, but the distance between them precludes π—π interaction. The pyridyl rings on both Schiff bases are almost orthogonal to each other at an angle of 85.06 (11)°.

The Zn^II^-donor atom bond lengths Zn—S [2.485 (6), 2.5263 (6) Å], Zn—N_py_ [2.2117 (17), 2.2692 (16) Å] and Zn—N_imine_ [2.1005 (16), 2.1032 (17) Å] compare well with a related octahedral Zn^II^ complex containing a pyridine-2-aldehyde Schiff base of S-benzyldithiocarbazate with Zn—N distances between 2.126 and 2.347 Å and Zn—S distances between 2.4514 and 2.4540 Å which are normal for multidentate bonding (Tarafder *et al.*, 2001). Although none of the bond angles in the complex conformed to the ideal values expected of a regular octahedral geometry, this appears to be common in six-coordinate metal complexes of Schiff base ligands derived from dithiocarbazic acid and is attributed to the restricted bite angles of the planar NNS tridentate ligands. (Ali *et al.*, 2001; Tarafder *et al.*, 2001). Weak C—H···S hydrogen bonds are observed and may consolidate the crystal packing (Fig. 2, Table 2).

Further background on the coordination chemistry of hydrazine carbodithioates is given by Ravoof *et al.* (2010).

### Experimental

Zinc(II) saccharinate, [Zn(sac)_2_(H_2_O)_4_]^.^2H_2_O was synthesized using a similar procedure as for the synthesis of Cu(II) saccharinate (Ravoof *et al.* (2004)). The title compound was synthesized following the procedure by Ali *et al.* (1997). The Schiff base was dissolved in acetonitrile (50 ml) and mixed with an equimolar quantity of zinc(II) saccharinate in acetonitrile (25 ml). The resulting mixture was heated on a water bath until the volume reduced to *ca* 30 ml. On standing overnight in the fridge, the mixture yielded yellow crystals suitable for X-ray analysis.

### Refinement

H atoms were all located in difference maps; those attached to carbon atoms were repositioned geometrically. The H atoms were initially refined with soft restraints on the bond lengths and angles to regularize their geometry (C—H in the range 0.93–0.98, N—H in the range 0.86–0.89 Å) and *U*_iso_(H)(in the range 1.2–1.5 times *U*_eq_ of the parent atom), after which the positions were refined with riding constraints.

### Crystal data

**Table:**

[Zn(C_15_H_14_N_3_S_2_)_2_]	*F*(000) = 1376
*M**_r_* = 666.24	*D*_x_ = 1.438 Mg m^−^^3^
Monoclinic, *P*2_1_/*c*	Mo *K*α radiation, λ = 0.71073 Å
Hall symbol: -P 2ybc	Cell parameters from 6730 reflections
*a* = 14.8931 (8) Å	θ = 2–29°
*b* = 13.0630 (5) Å	µ = 1.10 mm^−^^1^
*c* = 17.1706 (9) Å	*T* = 150 K
β = 112.855 (6)°	Prism, yellow
*V* = 3078.2 (3) Å^3^	0.24 × 0.18 × 0.16 mm
*Z* = 4	

### Data collection

**Table:**

Oxford Diffraction Gemini diffractometer	7140 independent reflections
Radiation source: sealed X-ray tube	5960 reflections with *I* > 2.0σ(*I*)
Graphite monochromator	*R*_int_ = 0.040
φ scans	θ_max_ = 28.9°, θ_min_ = 2.2°
Absorption correction: multi-scan (*CrysAlis PRO*; Agilent, 2011)	*h* = −19→16
*T*_min_ = 0.82, *T*_max_ = 0.84	*k* = −17→16
20496 measured reflections	*l* = −23→22

### Refinement

**Table:**

Refinement on *F*^2^	Primary atom site location: structure-invariant direct methods
Least-squares matrix: full	Hydrogen site location: difference Fourier map
*R*[*F*^2^ > 2σ(*F*^2^)] = 0.035	H-atom parameters constrained
*wR*(*F*^2^) = 0.076	Method = Modified Sheldrick *w* = 1/[σ^2^(*F*^2^) + (0.02*P*)^2^ + 2.2*P*], where *P* = (max(*F*_o_^2^,0) + 2*F*_c_^2^)/3
*S* = 1.00	(Δ/σ)_max_ = 0.001
7140 reflections	Δρ_max_ = 0.60 e Å^−^^3^
370 parameters	Δρ_min_ = −0.44 e Å^−^^3^
0 restraints	

### Special details

**Table:**

Experimental. The crystal was placed in the cold stream of an Oxford Cryosystems open-flow nitrogen cryostat (Cosier & Glazer, 1986) with a nominal stability of 0.1 K.Cosier, J. & Glazer, A.*M*., 1986. J. Appl. Cryst. 105–107.

### Fractional atomic coordinates and isotropic or equivalent isotropic displacement parameters (Å^2^)

**Table:**

	*x*	*y*	*z*	*U*_iso_*/*U*_eq_	
Zn1	0.300629 (18)	0.602950 (17)	0.084816 (14)	0.0209	
C101	0.27973 (16)	0.77374 (16)	−0.03043 (13)	0.0272	
N102	0.25268 (13)	0.74645 (13)	0.02846 (10)	0.0231	
N103	0.20970 (14)	0.81978 (13)	0.06093 (11)	0.0266	
C104	0.18607 (15)	0.78256 (15)	0.12131 (13)	0.0238	
S105	0.20083 (4)	0.66180 (4)	0.16361 (3)	0.0242	
S106	0.13026 (5)	0.86700 (4)	0.16857 (4)	0.0303	
C107	0.1375 (2)	0.99155 (16)	0.12297 (15)	0.0360	
C108	0.10104 (17)	1.06947 (15)	0.16809 (13)	0.0254	
C109	0.00675 (19)	1.10708 (19)	0.13364 (16)	0.0401	
C110	−0.0244 (2)	1.1781 (2)	0.1783 (2)	0.0518	
C111	0.0374 (2)	1.2108 (2)	0.25574 (18)	0.0475	
C112	0.1300 (2)	1.17467 (19)	0.29026 (16)	0.0419	
C113	0.16190 (18)	1.10461 (17)	0.24719 (14)	0.0329	
C114	0.32420 (16)	0.69782 (16)	−0.06656 (12)	0.0254	
N115	0.33418 (12)	0.60182 (13)	−0.03328 (10)	0.0220	
C116	0.36939 (15)	0.52813 (17)	−0.06804 (12)	0.0249	
C117	0.39613 (17)	0.54928 (19)	−0.13592 (13)	0.0323	
C118	0.38820 (17)	0.6466 (2)	−0.16798 (14)	0.0353	
C119	0.35103 (17)	0.72300 (19)	−0.13278 (13)	0.0318	
C120	0.37826 (17)	0.42206 (17)	−0.03333 (14)	0.0310	
C201	0.33127 (15)	0.39948 (15)	0.16182 (12)	0.0219	
N202	0.37232 (12)	0.48744 (12)	0.17189 (10)	0.0193	
N203	0.45554 (12)	0.50154 (12)	0.24379 (10)	0.0209	
C204	0.49345 (15)	0.59270 (15)	0.24628 (12)	0.0201	
S205	0.46105 (4)	0.68753 (4)	0.17153 (3)	0.0241	
S206	0.59395 (4)	0.62465 (4)	0.33815 (3)	0.0270	
C207	0.60458 (17)	0.51704 (16)	0.40886 (13)	0.0287	
C208	0.65317 (16)	0.55374 (16)	0.49885 (12)	0.0251	
C209	0.75115 (17)	0.53836 (17)	0.54403 (13)	0.0296	
C210	0.79462 (18)	0.5666 (2)	0.62843 (14)	0.0378	
C211	0.7396 (2)	0.6122 (2)	0.66756 (15)	0.0422	
C212	0.6416 (2)	0.6308 (2)	0.62209 (15)	0.0430	
C213	0.59881 (18)	0.60225 (18)	0.53807 (15)	0.0351	
C214	0.23803 (15)	0.38605 (15)	0.09042 (12)	0.0216	
N215	0.20544 (12)	0.46762 (13)	0.03850 (10)	0.0218	
C216	0.11911 (16)	0.46101 (17)	−0.02648 (13)	0.0266	
C217	0.06312 (18)	0.37251 (19)	−0.04065 (15)	0.0350	
C218	0.09638 (18)	0.28982 (19)	0.01244 (15)	0.0388	
C219	0.18571 (17)	0.29602 (17)	0.07917 (14)	0.0302	
C220	0.08524 (18)	0.55213 (19)	−0.08347 (14)	0.0369	
H1011	0.2715	0.8424	−0.0515	0.0327*	
H1072	0.2072	1.0050	0.1335	0.0461*	
H1071	0.0962	0.9913	0.0627	0.0454*	
H1091	−0.0363	1.0851	0.0796	0.0501*	
H1101	−0.0889	1.2035	0.1551	0.0624*	
H1111	0.0150	1.2586	0.2866	0.0587*	
H1121	0.1747	1.1974	0.3454	0.0517*	
H1131	0.2276	1.0814	0.2716	0.0400*	
H1171	0.4218	0.4964	−0.1593	0.0404*	
H1181	0.4076	0.6625	−0.2132	0.0415*	
H1191	0.3423	0.7915	−0.1540	0.0399*	
H1202	0.4177	0.3813	−0.0527	0.0470*	
H1201	0.4052	0.4216	0.0275	0.0468*	
H1203	0.3161	0.3906	−0.0510	0.0476*	
H2011	0.3592	0.3449	0.1992	0.0266*	
H2072	0.6431	0.4624	0.3966	0.0372*	
H2071	0.5394	0.4936	0.3977	0.0362*	
H2091	0.7893	0.5079	0.5173	0.0370*	
H2101	0.8626	0.5554	0.6590	0.0470*	
H2111	0.7694	0.6310	0.7260	0.0503*	
H2121	0.6039	0.6645	0.6492	0.0506*	
H2131	0.5299	0.6139	0.5059	0.0429*	
H2171	0.0017	0.3713	−0.0865	0.0417*	
H2181	0.0575	0.2294	0.0036	0.0463*	
H2191	0.2108	0.2407	0.1158	0.0359*	
H2202	0.0192	0.5429	−0.1229	0.0561*	
H2201	0.0899	0.6126	−0.0497	0.0562*	
H2203	0.1277	0.5617	−0.1133	0.0567*	

### Atomic displacement parameters (Å^2^)

**Table:**

	*U*^11^	*U*^22^	*U*^33^	*U*^12^	*U*^13^	*U*^23^
Zn1	0.02306 (14)	0.01875 (12)	0.02010 (12)	0.00037 (10)	0.00743 (10)	0.00014 (9)
C101	0.0334 (13)	0.0240 (11)	0.0244 (10)	0.0000 (9)	0.0116 (9)	0.0036 (8)
N102	0.0235 (10)	0.0233 (9)	0.0218 (8)	0.0011 (7)	0.0080 (7)	−0.0018 (7)
N103	0.0333 (11)	0.0199 (9)	0.0291 (9)	0.0036 (8)	0.0149 (8)	−0.0010 (7)
C104	0.0236 (11)	0.0212 (10)	0.0262 (10)	−0.0010 (9)	0.0092 (9)	−0.0057 (8)
S105	0.0270 (3)	0.0210 (3)	0.0275 (3)	−0.0008 (2)	0.0137 (2)	−0.0007 (2)
S106	0.0387 (3)	0.0215 (3)	0.0403 (3)	0.0000 (2)	0.0257 (3)	−0.0030 (2)
C107	0.0553 (17)	0.0231 (11)	0.0379 (13)	0.0020 (11)	0.0271 (12)	−0.0009 (10)
C108	0.0333 (13)	0.0169 (10)	0.0298 (11)	0.0009 (9)	0.0163 (10)	0.0021 (8)
C109	0.0324 (14)	0.0358 (14)	0.0423 (14)	−0.0022 (11)	0.0038 (11)	0.0000 (11)
C110	0.0314 (15)	0.0476 (16)	0.076 (2)	0.0186 (13)	0.0209 (15)	0.0102 (15)
C111	0.061 (2)	0.0376 (15)	0.0544 (17)	0.0121 (14)	0.0335 (15)	−0.0045 (12)
C112	0.0561 (18)	0.0335 (13)	0.0353 (13)	0.0042 (12)	0.0169 (12)	−0.0056 (11)
C113	0.0322 (13)	0.0313 (12)	0.0318 (12)	0.0081 (10)	0.0087 (10)	0.0022 (10)
C114	0.0250 (12)	0.0316 (11)	0.0182 (9)	−0.0017 (9)	0.0068 (8)	−0.0004 (8)
N115	0.0209 (9)	0.0264 (9)	0.0177 (8)	−0.0019 (7)	0.0064 (7)	−0.0024 (7)
C116	0.0175 (11)	0.0346 (12)	0.0209 (10)	−0.0013 (9)	0.0056 (8)	−0.0075 (9)
C117	0.0267 (12)	0.0474 (14)	0.0253 (11)	−0.0007 (11)	0.0128 (10)	−0.0083 (10)
C118	0.0314 (13)	0.0554 (16)	0.0219 (10)	−0.0043 (12)	0.0135 (10)	0.0011 (10)
C119	0.0343 (13)	0.0409 (13)	0.0214 (10)	−0.0023 (11)	0.0120 (10)	0.0039 (9)
C120	0.0313 (13)	0.0317 (12)	0.0323 (12)	0.0025 (10)	0.0150 (10)	−0.0060 (9)
C201	0.0264 (11)	0.0183 (10)	0.0214 (9)	−0.0001 (8)	0.0098 (9)	0.0004 (8)
N202	0.0214 (9)	0.0183 (8)	0.0188 (8)	−0.0004 (7)	0.0083 (7)	−0.0024 (6)
N203	0.0205 (9)	0.0215 (8)	0.0191 (8)	−0.0013 (7)	0.0061 (7)	−0.0005 (7)
C204	0.0193 (10)	0.0233 (10)	0.0187 (9)	0.0007 (8)	0.0084 (8)	−0.0014 (8)
S205	0.0259 (3)	0.0211 (2)	0.0236 (2)	−0.0033 (2)	0.0079 (2)	0.0020 (2)
S206	0.0254 (3)	0.0278 (3)	0.0226 (2)	−0.0076 (2)	0.0037 (2)	0.0013 (2)
C207	0.0316 (13)	0.0248 (11)	0.0248 (10)	−0.0040 (9)	0.0057 (9)	0.0027 (9)
C208	0.0284 (12)	0.0226 (10)	0.0229 (10)	−0.0041 (9)	0.0084 (9)	0.0014 (8)
C209	0.0291 (13)	0.0298 (12)	0.0292 (11)	0.0011 (10)	0.0106 (10)	−0.0059 (9)
C210	0.0304 (14)	0.0474 (15)	0.0294 (12)	−0.0009 (11)	0.0048 (10)	−0.0078 (11)
C211	0.0447 (16)	0.0558 (17)	0.0248 (11)	−0.0060 (13)	0.0123 (11)	−0.0100 (11)
C212	0.0444 (16)	0.0561 (17)	0.0357 (13)	0.0046 (13)	0.0233 (12)	−0.0062 (12)
C213	0.0287 (13)	0.0452 (14)	0.0332 (12)	0.0035 (11)	0.0140 (10)	0.0039 (11)
C214	0.0242 (11)	0.0216 (10)	0.0229 (10)	−0.0027 (8)	0.0134 (9)	−0.0041 (8)
N215	0.0232 (9)	0.0227 (9)	0.0213 (8)	−0.0010 (7)	0.0106 (7)	−0.0034 (7)
C216	0.0227 (11)	0.0341 (12)	0.0240 (10)	−0.0007 (9)	0.0101 (9)	−0.0047 (9)
C217	0.0258 (13)	0.0433 (14)	0.0315 (12)	−0.0091 (11)	0.0064 (10)	−0.0085 (10)
C218	0.0356 (15)	0.0367 (14)	0.0425 (14)	−0.0183 (11)	0.0136 (12)	−0.0071 (11)
C219	0.0328 (13)	0.0266 (11)	0.0310 (11)	−0.0065 (10)	0.0120 (10)	−0.0015 (9)
C220	0.0281 (13)	0.0416 (14)	0.0326 (12)	0.0022 (11)	0.0028 (10)	0.0027 (11)

### Geometric parameters (Å, º)

**Table:**

Zn1—N102	2.1032 (17)	C120—H1202	0.944
Zn1—S105	2.4885 (6)	C120—H1201	0.963
Zn1—N115	2.2692 (16)	C120—H1203	0.948
Zn1—N202	2.1005 (16)	C201—N202	1.281 (2)
Zn1—S205	2.5263 (6)	C201—C214	1.463 (3)
Zn1—N215	2.2117 (17)	C201—H2011	0.942
C101—N102	1.276 (3)	N202—N203	1.380 (2)
C101—C114	1.458 (3)	N203—C204	1.312 (2)
C101—H1011	0.956	C204—S205	1.713 (2)
N102—N103	1.384 (2)	C204—S206	1.752 (2)
N103—C104	1.311 (3)	S206—C207	1.824 (2)
C104—S105	1.715 (2)	C207—C208	1.507 (3)
C104—S106	1.758 (2)	C207—H2072	0.988
S106—C107	1.827 (2)	C207—H2071	0.964
C107—C108	1.503 (3)	C208—C209	1.376 (3)
C107—H1072	0.998	C208—C213	1.391 (3)
C107—H1071	0.977	C209—C210	1.388 (3)
C108—C109	1.385 (3)	C209—H2091	0.945
C108—C113	1.386 (3)	C210—C211	1.380 (3)
C109—C110	1.393 (4)	C210—H2101	0.953
C109—H1091	0.945	C211—C212	1.383 (4)
C110—C111	1.359 (4)	C211—H2111	0.958
C110—H1101	0.946	C212—C213	1.383 (3)
C111—C112	1.356 (4)	C212—H2121	0.962
C111—H1111	0.959	C213—H2131	0.970
C112—C113	1.373 (3)	C214—N215	1.353 (3)
C112—H1121	0.968	C214—C219	1.382 (3)
C113—H1131	0.953	N215—C216	1.338 (3)
C114—N115	1.362 (3)	C216—C217	1.390 (3)
C114—C119	1.383 (3)	C216—C220	1.498 (3)
N115—C116	1.342 (3)	C217—C218	1.376 (3)
C116—C117	1.398 (3)	C217—H2171	0.948
C116—C120	1.494 (3)	C218—C219	1.380 (3)
C117—C118	1.372 (3)	C218—H2181	0.955
C117—H1171	0.950	C219—H2191	0.936
C118—C119	1.389 (3)	C220—H2202	0.960
C118—H1181	0.951	C220—H2201	0.967
C119—H1191	0.956	C220—H2203	0.964
			
N102—Zn1—S105	78.87 (5)	C114—C119—H1191	119.7
N102—Zn1—N115	75.40 (6)	C116—C120—H1202	110.7
S105—Zn1—N115	150.83 (5)	C116—C120—H1201	111.9
N102—Zn1—N202	162.87 (6)	H1202—C120—H1201	109.0
S105—Zn1—N202	94.63 (5)	C116—C120—H1203	110.5
N115—Zn1—N202	113.82 (6)	H1202—C120—H1203	107.8
N102—Zn1—S205	87.46 (5)	H1201—C120—H1203	106.7
S105—Zn1—S205	99.92 (2)	N202—C201—C214	117.93 (18)
N115—Zn1—S205	92.49 (5)	N202—C201—H2011	121.7
N202—Zn1—S205	77.98 (5)	C214—C201—H2011	120.3
N102—Zn1—N215	119.43 (6)	Zn1—N202—C201	117.66 (14)
S105—Zn1—N215	90.44 (4)	Zn1—N202—N203	124.91 (12)
N115—Zn1—N215	90.36 (6)	C201—N202—N203	117.12 (16)
N202—Zn1—N215	76.08 (6)	N202—N203—C204	112.08 (16)
S205—Zn1—N215	152.73 (5)	N203—C204—S205	129.78 (16)
N102—C101—C114	118.85 (19)	N203—C204—S206	116.88 (15)
N102—C101—H1011	121.8	S205—C204—S206	113.33 (11)
C114—C101—H1011	119.3	Zn1—S205—C204	92.99 (7)
Zn1—N102—C101	117.28 (15)	C204—S206—C207	103.81 (10)
Zn1—N102—N103	123.99 (13)	S206—C207—C208	108.68 (14)
C101—N102—N103	117.62 (18)	S206—C207—H2072	108.8
N102—N103—C104	111.73 (17)	C208—C207—H2072	111.1
N103—C104—S105	130.28 (16)	S206—C207—H2071	107.0
N103—C104—S106	116.92 (15)	C208—C207—H2071	110.5
S105—C104—S106	112.80 (12)	H2072—C207—H2071	110.6
Zn1—S105—C104	93.40 (7)	C207—C208—C209	120.9 (2)
C104—S106—C107	104.10 (10)	C207—C208—C213	120.2 (2)
S106—C107—C108	106.78 (15)	C209—C208—C213	118.9 (2)
S106—C107—H1072	108.1	C208—C209—C210	120.8 (2)
C108—C107—H1072	110.3	C208—C209—H2091	119.5
S106—C107—H1071	109.5	C210—C209—H2091	119.6
C108—C107—H1071	110.0	C209—C210—C211	119.9 (2)
H1072—C107—H1071	112.1	C209—C210—H2101	119.9
C107—C108—C109	122.0 (2)	C211—C210—H2101	120.2
C107—C108—C113	120.0 (2)	C210—C211—C212	119.8 (2)
C109—C108—C113	118.0 (2)	C210—C211—H2111	119.9
C108—C109—C110	120.0 (2)	C212—C211—H2111	120.3
C108—C109—H1091	120.0	C211—C212—C213	119.9 (2)
C110—C109—H1091	120.0	C211—C212—H2121	119.5
C109—C110—C111	120.3 (2)	C213—C212—H2121	120.6
C109—C110—H1101	120.2	C208—C213—C212	120.6 (2)
C111—C110—H1101	119.5	C208—C213—H2131	118.8
C110—C111—C112	120.4 (2)	C212—C213—H2131	120.6
C110—C111—H1111	119.8	C201—C214—N215	116.03 (17)
C112—C111—H1111	119.8	C201—C214—C219	121.28 (19)
C111—C112—C113	120.2 (2)	N215—C214—C219	122.66 (19)
C111—C112—H1121	121.1	Zn1—N215—C214	112.14 (13)
C113—C112—H1121	118.7	Zn1—N215—C216	128.71 (14)
C108—C113—C112	121.1 (2)	C214—N215—C216	118.77 (18)
C108—C113—H1131	119.7	N215—C216—C217	121.1 (2)
C112—C113—H1131	119.2	N215—C216—C220	117.64 (19)
C101—C114—N115	116.19 (18)	C217—C216—C220	121.3 (2)
C101—C114—C119	120.8 (2)	C216—C217—C218	120.0 (2)
N115—C114—C119	123.0 (2)	C216—C217—H2171	118.6
Zn1—N115—C114	110.05 (13)	C218—C217—H2171	121.4
Zn1—N115—C116	131.44 (14)	C217—C218—C219	119.1 (2)
C114—N115—C116	118.14 (17)	C217—C218—H2181	120.2
N115—C116—C117	121.1 (2)	C219—C218—H2181	120.7
N115—C116—C120	118.50 (18)	C214—C219—C218	118.4 (2)
C117—C116—C120	120.42 (19)	C214—C219—H2191	120.5
C116—C117—C118	120.6 (2)	C218—C219—H2191	121.2
C116—C117—H1171	120.0	C216—C220—H2202	110.7
C118—C117—H1171	119.4	C216—C220—H2201	109.2
C117—C118—C119	118.6 (2)	H2202—C220—H2201	110.0
C117—C118—H1181	121.6	C216—C220—H2203	109.1
C119—C118—H1181	119.8	H2202—C220—H2203	110.1
C118—C119—C114	118.6 (2)	H2201—C220—H2203	107.7
C118—C119—H1191	121.7		

### Hydrogen-bond geometry (Å, º)

**Table:**

*D*—H···*A*	*D*—H	H···*A*	*D*···*A*	*D*—H···*A*
C217—H2171···S105^i^	0.95	2.82	3.697 (3)	155

Symmetry code: (i) −*x*, −*y*+1, −*z*.
